# A Novel Insight of Effects of a 3-Hz Binaural Beat on Sleep Stages During Sleep

**DOI:** 10.3389/fnhum.2018.00387

**Published:** 2018-09-24

**Authors:** Nantawachara Jirakittayakorn, Yodchanan Wongsawat

**Affiliations:** Brain Computer Interface Laboratory, Department of Biomedical Engineering, Faculty of Engineering, Mahidol University, Salaya, Thailand

**Keywords:** binaural beat, sleep modulation, delta activity, sleep stage, slow wave sleep

## Abstract

The dichotic presentation of two almost equivalent pure tones with slightly different frequencies leads to virtual beat perception by the brain. In this phenomenon, the so-called binaural beat has a frequency equaling the difference of the frequencies of the two pure tones. The binaural beat can entrain neural activities to synchronize with the beat frequency and induce behavioral states related to the neural activities. This study aimed to investigate the effect of a 3-Hz binaural beat on sleep stages, which is considered a behavioral state. Twenty-four participants were allocated to experimental and control groups. The experimental period was three consecutive nights consisting of an adaptation night, a baseline night, and an experimental night. Participants in both groups underwent the same procedures, but only the experimental group was exposed to the 3-Hz binaural beat on the experimental night. The stimulus was initiated when the first epoch of the N2 sleep stage was detected and stopped when the first epoch of the N3 sleep stage detected. For the control group, a silent sham stimulus was used. However, the participants were blinded to their stimulus group. The results showed that the N3 duration of the experimental group was longer than that of the control group, and the N2 duration of the experimental group was shorter than that of the control group. Moreover, the N3 latency of the experimental group was shorter.

## Introduction

**FIGURE 1:**
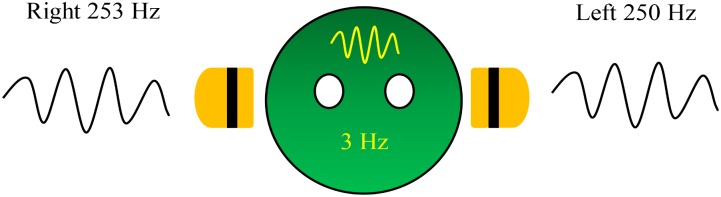
A 3-Hz binaural beat on a 250-Hz carrier tone generated in the brain.

Beat is a phenomenon that occurs by interference of two almost equivalent sinusoidal tones but with slightly different frequencies and presents as fluctuation of a single tone. The fluctuation single tone is perceived as amplitude modulation with the frequency equaling the difference of the frequencies of the two interfering tones. A binaural beat, which occurs when the two mentioned tones are simultaneously presented to different ears, a virtual fluctuation of a single tone is generated in the brain by the ascending auditory pathway. For example, when a sinusoidal 250-Hz pure tone is presented to the left ear, and a 253-Hz tone is simultaneously presented to the right ear, a fluctuation of amplitude with a frequency rate of 3 Hz is perceived by the brain (**Figure 1**). The binaural beat cannot be measured by measurement tools but can be perceived by humans because the origination of the beat is in the brain; however, the difference of the two presented frequencies is greater than 35 Hz or two separate tones would not be perceived (Oster, 1973). Moreover, one study measured perception of the beat on different frequency carrier tones – the tone that is generated at each ear – and suggested that an intermediate frequency carrier tone of 440 Hz facilitated a wider range of beat perception than lower or higher frequency carrier tones (Licklider et al., 1950). The auditory signal from each ear is conducted ipsilaterally along the ascending auditory pathway to the auditory cortex. However, at the brainstem, auditory signals from both sides are passed to the superior olivary complex (Kuwada et al., 1979; Schwarz and Taylor, 2005), the first nucleus in the ascending auditory pathway receiving bilateral auditory signals. The so-called binaural beat is spontaneously generated with a frequency equaling the difference of the frequencies of the two tones. The binaural beat is then conducted to the auditory cortex in phase-locked fashion described by the response of the inferior colliculus – a part of the ascending auditory pathway – as a binaural beat (Kuwada et al., 1979; McAlpine et al., 1996; Spitzer and Semple, 1998; Schwarz and Taylor, 2005; Karino et al., 2006).

After the primary auditory cortex receives the binaural beat signal, the signal is consequently sent to associated auditory areas and other associated areas inducing the brain to oscillate at the rate of binaural beat frequency, which can be measured by EEG rhythms. This phenomenon is called entrainment of neural oscillation (Wahbeh et al., 2007). EEG is a non-invasive technique for monitoring and recording electrical activity of the brain. Normally, spectral elements of EEG signals can be divided into five frequency bands: delta (0.5–4 Hz), theta (4–7 Hz), alpha (7–13 Hz), beta (13–30 Hz), and gamma (30–50 Hz) bands. These neural activities are entrained due to the frequency of the binaural beat, which is known as a frequency-following response (Worden and Marsh, 1968; Moushegian et al., 1973; Grose and Mamo, 2012). However, several studies have reported that binaural beats have effects on both EEG rhythms and psychophysiological aspects (Schwarz and Taylor, 2005; Pratt et al., 2009; Pratt et al., 2010; Lavallee et al., 2011; Chakalov et al., 2014).

The frequency-following response, which is one effect of a binaural beat, is described by the fact that binaural beat can entrain neurons to oscillate at the frequency rate related to the binaural beat frequency. Several studies have shown an entrainment effect in almost all frequency bands, including theta, beta, and gamma bands, after participants are exposed to a binaural beat within the ranges of those frequencies (Schwarz and Taylor, 2005; Draganova et al., 2008; Pratt et al., 2009; Magezi and Krumbholz, 2010; Ross et al., 2014). However, the cortical positions responding to the binaural beat remain a topic of discussion because different brain processes occur for different frequency activities (Ross et al., 2014), i.e., the cerebral cortex can be synchronized with a binaural beat, but previous studies have utilized different binaural stimuli in terms of carrier tone and beat frequency. Hence, with a phase-locked response of the ascending auditory pathway, different responses at the cortical level may appear. However, based on this discussion, other researchers have conducted experiments on the psychophysiological effects of binaural beats and shown interesting supporting evidence (Lane et al., 1998; Padmanabhan et al., 2005; Lavallee et al., 2011; Reedijk et al., 2013).

Normally, each EEG rhythm is associated with a particular activity, cognition, or behavior. For example, gamma oscillations are associated with the maintenance of arousal of the brain during the awake stage (Gray and Singer, 1989; Llinas and Pare, 1991; Gray, 1999; Vanderwolf, 2000), while theta oscillations are found during meditation (Takahashi et al., 2005; Lagopoulos et al., 2009) and associated with alertness, attention, orientation, and working memory, including the enhancement of cognitive and perceptual performances (Aftanas and Golocheikine, 2001; Stern et al., 2001). One study stated that listening to a 6-Hz binaural beat can induce EEG rhythms to reflect a meditative state and reduce negative emotions (Jirakittayakorn and Wongsawat, 2017a). Moreover, a binaural beat in the theta range also showed an effect on reducing stress and anxiety in pre-operative procedures (Padmanabhan et al., 2005), and a binaural beat in the beta range enhanced vigilance performance (Lane et al., 1998). A theta binaural beat could potentially be used to induce novice meditators into a deep meditative stage, while a beta binaural beat strongly inhibits meditators to maintain the same level of meditation (Lavallee et al., 2011). Recent studies have also shown that a binaural beat in the beta range improves word recall and affects long-term memory (Garcia-Argibay et al., 2017), and a binaural beat in the gamma range enhances short-term memory (Jirakittayakorn and Wongsawat, 2017b). These studies support an entrainment of neural oscillations in the brain because these activities, cognitions, and behaviors are related to specific neural oscillations that are specifically induced and entrained in EEG rhythms.

From literature, binaural beats have been shown to entrain neural oscillations, even though obviously cortical areas responding to the binaural beat remain under discussion. Moreover, activities, cognitions, and behaviors can be manipulated via those entrainments following the perception of a binaural beat and presented depending on the related EEG rhythm. This evidence suggests that binaural beats affect neural activities, as shown in EEG recordings, and thus, binaural beats could be utilized to modulate behavioral states. However, observation of responses of binaural beat in range of delta activity is lacking because delta activity is typically found during sleep in normal adults.

Sleep is a physiological activity and is considered as one of the natural states of the body, occurring in every species. It is a behavioral state that can be described by reduced motility and sensory responses to external stimuli (Carskadon and Dement, 2005). In short, all sensory thresholds during sleep are higher than during the awake stage. Sleep and wakefulness are regulated by the ascending arousal system, which originates in the brainstem, sending projection fibers throughout the thalamus, hypothalamus, basal forebrain, and cerebral cortex. Several nuclei population groups are included in this system (Schwartz and Roth, 2008). Among those nuclei groups, some are categorized as part of the sleep-promoting system (Sherin et al., 1996; Szymusiak et al., 1998; Gallopin et al., 2000; Chamberlin et al., 2003), and some are firing as wake promoting system (Moruzzi and Magoun, 1949; Panula et al., 1984; Takahashi et al., 2006). These two groups of nuclei play regulatory roles as a flip-flop switch (Saper et al., 2001, 2005, 2010; Lu et al., 2006), with reciprocal interactions to each other. The reciprocal flip-flop switch is essential to prevent a prolonged transitional stage between sleep and awake stages (Saper et al., 2010). In other words, nuclei in the wake-promoting system fire to inhibit nuclei in the sleep-promoting system to maintain the waking state, and vice versa; nuclei in the sleep-promoting system also inhibit nuclei in the wake-promoting system during sleep, leading to different neural oscillations that can be observed by changes in EEG signals during sleep.

The different EEG patterns during sleep are indicated as sleep stages. Sleep stages have been classified into two types by eye movement which are non-rapid eye movement and rapid eye movement sleep. For non-rapid eye movement (NREM), three different EEG patterns are indicated by the American Academy of Sleep Medicine (AASM), which are N1, N2, and N3 stages (Iber et al., 2007; Berry et al., 2015). The patterns of EEG signals are as follows: N1 shows low voltage with a mixed frequency of EEGs; N2 shows sleep spindle activity with an average frequency of 14 Hz and the K-complex, a brief negative high-voltage peak followed by a slower positive complex; N3 shows many slow waves or delta waves in EEGs. Rapid eye movement (REM) shows similar EEG pattern with the N1 stage during which random and rapid movement of the eyes also occurs. These patterns of EEG repeatedly occur during the sleep period in a cyclical fashion. Sensory thresholds are higher during sleep than during the awake stage, and responses to external stimuli are decreased; however, only one sensation that is active throughout the sleep period is auditory sensation. The auditory system is the only system being active during sleep, with a lower threshold than other sensory systems, and plays a role as a guardian to prevent against danger by screening all audible sounds in the surrounding environment (Velluti, 1997). The threshold of auditory stimuli is approximately 60 dB SPL (Williams et al., 1964), and sound intensity over 65 dB SPL induces an arousal response (Velluti, 1997). Each sleep stage responds differently to the same auditory stimulus intensity. The N2 stage shows an obvious response and less variation, while the response is not observed during the N3 stage (Velluti, 1997). Therefore, binaural beats could be used to modulate sleep stages, as they can be used to modulate behavioral states followed by an entrainment effect. Moreover, as there is a lack of investigations of the effects of binaural beats during sleep, we present important, novel observations of their effects during sleep.

One another interesting point of sleep is memory. Once information is received in the first time during learning period, that memory is labile and fragile, after a period of time, that memory is consolidated and becomes long-lasting memory during sleep, especially during N3 stage. However, due to modern lifestyle in nowadays, slow wave sleep or N3 stage seems to be disturbed by distress (Buckley and Schatzberg, 2005). The sleep is changed by reduction of slow wave sleep, REM sleep, delta power (Rolls et al., 2010), and sleep efficiency including increase in awakening (Kim and Dimsdale, 2007). With these reasons, memory consolidation during sleep is impaired due to N3 sleep stage reduction (Prince and Abel, 2013; Havekes et al., 2016). Increase in N3 sleep stage or slow wave sleep can be implied to improve strengthening memory in a person who lack of N3 sleep stage due to memory consolidation process, if N3 sleep stage can be modulated by the stimulus.

This study aimed to investigate the effect of a 3-Hz binaural beat stimulus on a 250-Hz carrier tone of 60 dB SPL on sleep stages, especially on stages N2 and N3. According to sleep characteristics, the N3 stage presents delta activity on EEGs and, thus, should be entrained by a 3-Hz binaural beat, which is in the range of delta activity, if the binaural beat is able to induce EEG rhythms to synchronize with its frequency. Therefore, the N3 stage should exhibit extended duration and shortened latency. Moreover, N3 stage play an important role in memory consolidation so increase in N3 sleep stage may lead to improve memory.

## Materials and Methods

This study aimed to investigate the effect of 3-Hz binaural beat on 250-Hz carrier tone on sleep stages by delivering binaural beat stimulus to participants in experimental group during the third night, experimental night. Electroencephalograms (EEGs), electro-oculograms (EOGs), and electromyograms (EMGs) were utilized to score sleep stages as recommended by the American Academy of Sleep Medicine (AASM) (Iber et al., 2007; Berry et al., 2015). Effect of the stimulus to sleep stage was focusly observed within subjects in experimental group; however, experimental and control groups were included in this study to confirm stimulus effect not habituation to environment. All experimental procedures were approved by the Institutional Review Board of Mahidol University with certificate of approval (COA) number 2016/119.1209. Prior to including the participants in the study, all procedures were described to the participants and written informed consent were given by all participants. The participants can freely withdraw their participations from the study by any reasons.

### Experimental Room

The experimental room was a sound-attenuated room and completely dark after turning off the lights with the temperature controlled to 25°C. The color of the room’s walls was ivory to promote neutral perceptions of emotion and mood according to white color (Sroykham et al., 2014) but without refulgent light reflection to arouse awakening. The bed was set under an air-conditioner to prevent air directly flowing onto the participant’s face and inducting congestion or allergies but allowing temperature to be controlled. A monitoring unit was located outside the experimental room for privacy, but video monitoring was available for monitoring the inside of the experimental room.

### Binaural Beat Stimulus

The stimulus used in this study was a 3-Hz binaural beat on a 250-Hz carrier tone, which was specifically generated for the experiment. A 250-Hz carrier tone was presented to the left ear and a 253-Hz tone was presented to the right ear. This binaural beat file was set to 60 dB SPL as the threshold of auditory stimulation during sleep (Williams et al., 1964).

### Participants

Twenty-four healthy participants with an average age of 24.12 years and a standard deviation of 2.54 years were included in this study (13 males and 11 females). The participants were randomly divided into two groups, experimental and control groups. The purpose of this study and the experimental procedures were explained to all participants before participation, but participants were blinded to the details of the stimulus and group allocation. The experimental group was composed of 16 participants, eight males and eight females, with an average age of 24.75 years and a standard deviation of 1.92 years, while the control group was composed of eight participants, five males and three females, with an average age of 22.88 years and a standard deviation of 3.27 years. However, one participant of the experimental group could not complete the experimental protocol on the third night because of a personal issue unrelated to the experimental procedures, and therefore, he was excluded from the study. Finally, 15 participants of the experimental group were included in the statistical analysis.

### Sleep Parameter Recording

Electroencephalogram, EOG, and EMG were utilized to score sleep stages in this study, as recommended by the AASM. These bio-signals were used without full polysomnography (PSG) in this study because they are sufficient for scoring sleep stages and ensuring comfort to the participants. Four EEG electrodes were attached to positions of F4, C4, O2, and Cz due to the international 10/20 system for EEG electrode placement. These positions were practically recommended for sleep stage scoring by the AASM standard (Iber et al., 2007; Berry et al., 2015). A reference EEG electrode was placed at the left mastoid process (M1 position), while a ground electrode was placed at the right mastoid process (M2 position). For EOG, an electrode was attached to the left and right sides, 1 cm below the left eyelid and 1 cm above the right eyelid. A reference EOG electrode was placed on the forehead. Two electrodes for chin EMG were placed as mental electrode and submental electrode. The mental electrode was placed at the midline 1 cm above the inferior edge of the mandible, and the submental electrode was placed at the midline 2 cm below the inferior edge. A ground electrode used for both EOG and EMG was placed at the same position as EEG on the right mastoid process (M2 position). Both EOG and EMG were used to determine REM stage.

All EEG, EOG, and EMG were recorded using a Guger Technologies (g.tec) G.USBamp 3.0 16 channels Bio-signal amplifier brain computer interface BCI2000 as amplifier for data acquisition with a sampling rate of 516 samples per second, which is higher than the recommendation of the AASM of 500 samples per second. All signals were sampled at the same rate, but different band-pass filters were used as follows: 0.3 to 35 Hz for all EEG and EOG channels, and 10 to 100 Hz for chin EMG.

After wiring all electrodes, the system was connected to a monitoring PC via a networking system which displayed 30 s per one epoch to monitor the real-time sleep stage with video monitoring outside the experimental room.

### Experimental Procedures

The experiment was composed of three consecutive nights, corresponding to adaptation, baseline, and experimental nights. All participants were required to attend all consecutive nights for inclusion in the statistical analysis. Both experimental and control groups underwent recordings during these three consecutive nights, and all procedures were the same. The last night, the experimental night, differed between the experimental and control groups. On this night, the 3-Hz binaural beat stimulus was delivered to the experimental group, while a sham stimulus was presented to the control group. The sham stimulus used in this study was silence for comparing sleep stages between a binaural beat and a non-binaural beat.

For each night of the experiment period, the participant arrived at the experimental room at 9 p.m. to prepare themselves for participation in the study. The procedures were described to the participants on the first night, the adaptation night, at which time the participants completed the informed consent form. Participants can withdraw their participations for any reasons. Emotion and mood were then evaluated by the Brunel Mood Scale (BRUMS) questionnaire.

The BRUMS is a self-report emotional state questionnaire composed of 24 items. These items correspond to a 6-factor model including “Anger,” “Confusion,” “Depression,” “Fatigue,” “Tension,” and “Vigor.” Each item is responded to using a 5-point Likert scale, which ranges from 0 to 4 representing “not at all” to “extremely” depending of the participant’s feelings. However, only fatigue and vigor factors were focus in this study to indicate sleep disturbance in the last night.

After completing the emotional questionnaire, the participant put on a pair of Beats^x^ wireless in-ear headphones. The 3-Hz binaural beat stimulus was delivered to each participant for 3 s to test the sound, and then, a sound was separately delivered to each ear for another 3 s each. The headphones were then removed. The participant’s head was measured for electrode positions according to the international 10/20 system. F4, C4, O2, and Cz positions were marked. Genuine Grass^®^ 10 mm gold cup electrodes were utilized for bio-signal acquisition with Ten20^®^ EEG conductive paste. Prior to attaching each electrode at their positions, the skin was gently scrubbed to remove dead cells and the sebum layer for close attachment between the electrode and skin. All eleven electrodes were attached as described in the sleep parameter recording section and connected to the g.tec amplifier and monitoring PC. The headphones were put back on and gently attached to the participant’s ears by tape to prevent the headphones from falling out of the ears during the night. The participant subsequently went to bed and the lights were turned off at 11 p.m. At 7 a.m. of the following morning, the participant was awakened by turning on the lights, and all electrodes were removed. During the sleep period, EEG, EOG, and EMG signals were continuously monitored. The BRUMS was completed again by the participant to evaluate their emotional state. The appointment for the next night was scheduled at the same time of the previous night.

**FIGURE 2:**
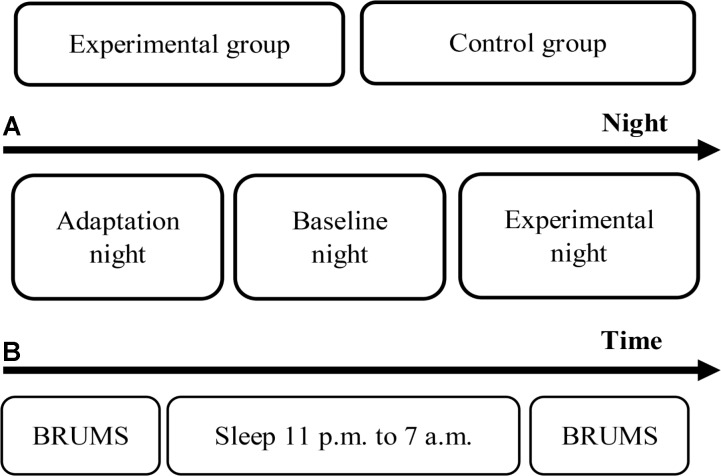
Experimental procedures. **(A)** Timeline of the experimental period. **(B)** Timeline of each night.

These procedures were conducted for each participant both in the experimental and control groups for all study nights. For the last night of the experimental group (the experimental night), a 3-Hz binaural beat was initiated for delivery to the participants each time that the first epoch of N2 stage appeared and was stopped when the first epoch of N3 stage appeared. The auditory stimulation was continually delivered to the participants although N1, and REM sleep stages appeared. The reason of delivery stimulus during N2 was that the N2 stage shows an obvious response and less variation, while the response is not observed during the N3 stage (Velluti, 1997). Moreover, one study presented audible sound with intensity around 68 dB SPL to participants who slept in N2 sleep stage, and that sound let the participants waking up (Bonnet, 1986). More recent studies on auditory stimulation during sleep have delivered auditory stimulus to the participants during N2 sleep stage or deeper appeared by fixing time (Ngo et al., 2013), or at slow oscillation was detected during N2 sleep stage or deeper (Besedovsky et al., 2017). However, stable N2 sleep stage is utilized as an important stimulus trigger criterion. Furthermore, the primary focus sleep stage in this study was N3 sleep stage, and transition from N2 sleep stage to N3 sleep stage typically showed more possibility compared to transition from other sleep stages to N3 sleep stage by releasing slow oscillation stimulus to sleepers during sleep (Saebipour et al., 2015). Therefore, providing the stimulus during N2 stage can investigate less variation of response, and is consistent with several studies. These procedures were continued through the 5th hour of sleep, after which the stimulus was terminated. As mentioned, human sleep stages are cyclical, and therefore, the 3-Hz binaural beat stimulus was stopped if a waking stage appeared for three consecutive epochs after releasing. The experimental procedures are shown in **Figure 2**.

### Sleep Stage Scoring

Sleep stages of the recorded data were scored for every epoch following the AASM standards for sleep stage scoring as W, N1, N2, N3, and REM stages, depending on the characteristics of EEG, EOG, and EMG signals (Iber et al., 2007; Berry et al., 2015). The sleep parameters focused on in this study were total sleep time (TST), sleep latency (SL), REM latency, arousal index, wake after sleep onset (WASO), percent of sleep efficiency, time in each stage, percent of TST in each stage, N3 latency, N3 duration, and N2 duration. Three scorers were included in scoring process, each scorer scored all participants data in one of three consecutive nights, but the night was blinded. Arousal in this study is scored due to AASM scoring manual and includes movement arousal. Arousal index is then calculated from total arousal divided by TST.

### EEG Data Analysis

Electroencephalogram signals recorded along the baseline and the experimental nights, night 2 and night 3, were processed to analyze in frequency domain by fast Fourier transform (FFT). Waking stage and arousal including movement arousal were removed from the analysis process because waking stage was not associated with increase in delta activity during sleep induced by 3-Hz binaural beat, while movement arousal signal was transformed into low frequency data in frequency domain, especially in delta activity. Other noise signals were also excluded from the analysis such as electrode displacement signal. Absolute power of each frequency was summed to represent absolute power of each frequency band including delta (0.5–4 Hz), theta (4–7 Hz), alpha (7–13 Hz), beta (13–30 Hz), and gamma (30–50 Hz) bands. This process was conducted on every electrode positions which were F4, C4, O2, and Cz. Consequently, average of four electrode positions was conducted in each frequency band to represent that frequency band of each participant during night 2 and night 3 for statistical analysis.

Another FFT was performed on EEG signals in which the epoch was considered as N2 sleep stage in both experimental and control groups of night 2 and night 3 to reveal characteristics of delta activity in each condition. Absolute power of delta activity of N2 sleep stage was summed and used as delta power during N2 sleep stage of each group and night. Changes in delta power during N2 sleep stage in night 3 with respect to night 2 of each group were operated for correlation analysis.

### Statistical Analysis

Comparisons were performed for each mentioned parameter both within groups to investigate effect of 3-Hz binaural beat on sleep stage and between groups to confirm that occurred effect was the stimulus effect not habituation to environment. Paired *t*-tests were conducted to evaluate the effect of the 3-Hz binaural beat stimulus on the experimental night (night 3) and the baseline night (night 2) of both groups. The adaptation night (night 1) was compared with night 2 by paired *t*-tests to evaluate the effect of sleep deprivation due to the environmental changes. Independent *t*-tests were conducted to ensure that the observed effects were only due to the 3-Hz binaural beat stimulus and not to habituation to the changed environment. In other words, differences in parameters between night 3 and night 2 (d of night 3 and that of night 2 by paired *t*-tests) were consequently compared. *P*-values less than 0.05 were considered significant in all statistical analyses.

Similar comparisons were performed on FFT absolute power to compare each frequency band which were delta, theta, alpha, beta, and gamma bands both within groups and between groups. The analysis of frequency bands was conducted to investigate effect of 3-Hz binaural beat on neural activity during sleep and to evaluate association between N3 sleep stage and delta activity.

Correlation analysis via Fisher transformation was conducted to change in delta activity during N2 sleep stage of night 3 with respect to night 2 in the group, that had expressed significant difference in delta activity within the whole night by paired *t*-test, and N3 duration and N3 latency to evaluate the relationship between change in delta activity during auditory stimulation and the two primary variable investigated in this study, N3 duration and N3 latency. In other words, the correlation analysis was performed on delta activity during N2 sleep stage and N3 duration and N3 latency only in experimental group. *P*-value of less than 0.05 was considered significant.

Survival analysis with log-rank test was conducted on transition period from N2 sleep stage to N3 sleep stage to assess sleep fragmentation described somewhere (Swihart et al., 2015) between experimental and control groups only in night 3 due to 3-Hz binaural beat stimulation.

Paired *t*-tests were conducted on BRUMS score in both groups comparing between before sleep and after waking for night 3, only the focusing emotion which were fatigue and vigor factors to evaluate whether the stimulus disturbed the sleep period and which emotional states were significantly changed, either increased or decreased, in each group.

## Results

The stimulus was presented to participants in each group during the experimental night (night 3) of three consecutive nights. A 3-Hz binaural beat, which is in the delta range, was delivered to participants of the experimental group when the first epoch of the N2 stage appeared and was terminated when the first epoch of the N3 stage appeared; no sounds were delivered to the control group throughout the night. All participants were blinded to the experimental and control groups and underwent in the same procedures every night. Sleep stages and emotions were investigated following the stimulation.

### Sleep Parameters

**FIGURE 3:**
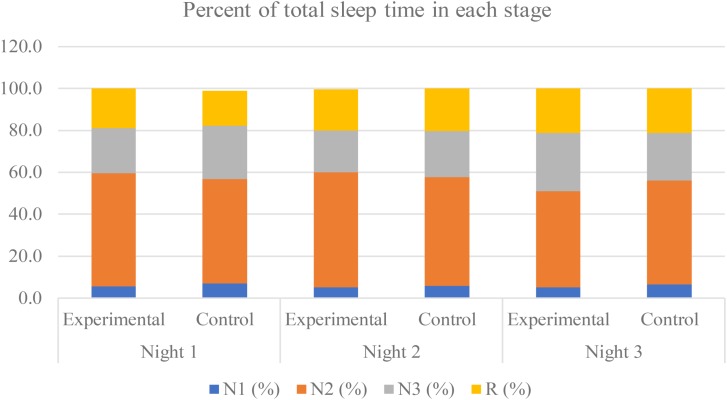
Percent of total sleep time in each stage during the experimental period of both groups. The non-rapid eye-movement stage 1, stage 2, stage 3, and the rapid-eye movement (REM) are labeled as “N1,” “N2,” “N3,” and “R,” respectively.

All sleep parameters of both the experimental group and the control group are shown in **Table 1**. The TST was approximately 450 min each night, with the maximum duration in the N2 stage of approximately 250 min, which was 50% of the TST. The percent of time in each stage is displayed in **Figure 3**.

**Table 1:** Averages of the sleep parameters of the experimental and control groups during the experimental period (mean ± SD).

Parameter	Experimental group			Control group		
	Night 1	Night 2	Night 3	Night 1	Night 2	Night 3
Total sleep time (min)	434.8 ± 41.3	457.4 ± 16.2	455.9 ± 19.1	446.7 ± 40.7	463.0 ± 8.0	456.7 ± 12.6
Sleep latency (min)	12.2 ± 10.8	8.8 ± 7.1	8.9 ± 9.4	6.2 ± 3.7	6.8 ± 2.8	9.1 ± 4.6
REM latency (min)	146.1 ± 86.9	95.1 ± 31.8	88.8 ± 30.5	103.8 ± 45.7	96.1 ± 41.3	87.8 ± 13.0
N3 latency (min)	21.0 ± 9.5	22.9 ± 19.6	13.5 ± 4.9	17.7 ± 5.8	17.6 ± 4.8	18.4 ± 5.6
Arousal index	3 ± 1.3	2 ± 1.1	2 ± 0.9	2 ± 0.7	2 ± 0.5	2 ± 0.8
Wake after sleep onset (min)	33.4 ± 38.7	14.3 ± 13.8	15.7 ± 13.9	27.6 ± 40.5	10.7 ± 6.6	14.8 ± 8.8
Sleep efficiency (%)	90.6 ± 8.6	95.3 ± 3.4	95.0 ± 4.0	93.1 ± 8.5	96.5 ± 1.7	95.1 ± 2.6
Time in N1 (min)	23.7 ± 12.0	23.7 ± 9.6	23.1 ± 11.3	30.6 ± 8.0	27.5 ± 7.8	29.6 ± 5.8
Time in N2 (min)	234.1 ± 29.2	251.0 ± 16.8	209.8 ± 42.8	222.8 ± 26.5	239.9 ± 24.6	226.9 ± 27.0
Time in N3 (min)	94.8 ± 27.7	90.6 ± 16.3	125.9 ± 30.8	207.3 ± 32.9	101.4 ± 28.3	103.1 ± 19.8
Time in REM (min)	82.3 ± 15.6	90.3 ± 21.4	97.0 ± 17.7	74.8 ± 23.6	94.2 ± 16.5	97.1 ± 18.2
Percent of N1 (%)	5.7 ± 3.3	5.2 ± 2.1	5.1 ± 2.6	7.0 ± 102	5.9 ± 1.7	6.5 ± 1.2
Percent of N2 (%)	53.9 ± 5.0	54.9 ± 3.7	45.9 ± 8.9	49.9 ± 4.2	51.9 ± 5.7	49.7 ± 5.8
Percent of N3 (%)	21.6 ± 5.1	19.8 ± 3.5	27.7 ± 6.9	25.4 ± 6.6	21.9 ± 6.1	22.6 ± 4.6
Percent of REM (%)	18.9 ± 3.1	19.7 ± 4.4	21.3 ± 3.7	16.6 ± 4.6	20.3 ± 3.3	21.2 ± 3.7

#### Experimental Group

The results show that in experimental period, participants almost slept in the N2 stage every night. The significant findings of the experimental group are shown in **Table 2**. Comparing the experimental night with the baseline night (night 3 compared with night 2), after the stimulus was released to the participants during sleep, the N3 sleep stage showed significant increases in terms of both minutes and percent (*p*-value <0.0001.) The N3 latency was significant shorter. The N2 sleep stage also showed significant decreases in terms of both minutes and percent (*p*-value <0.0001). Other sleep parameters did not show any significant differences. Average stimulation duration with standard deviation of experimental group in night 3 was 193.21 ± 35.27 min.

**Table 2:** Paired *t*-tests of sleep parameters between night 2 and night 3 of both the experimental and control groups; and independent *t*-tests of the mean difference between the experimental and control groups (comparison of d of night 2 and night 3 between groups; an up arrow indicates an increase or longer duration, and a down arrow indicates a decrease or shorter duration; ^∗^indicates a two-tailed test).

Parameter	Paired *t*-test						Independent *t*-test	
	Experimental group			Control group			*P*-value	Sig.
	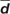 ± SD	*P*-value	Sig.	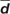 ± SD	*P*-value	Sig.		
Total sleep time (min)	-1.5 ± 24.2	0.8135	n.s.^∗^	-6.3 ± 10.3	0.1258	n.s.^∗^	0.5123	n.s.^∗^
Sleep latency (min)	0.1 ± 11.3	0.9820	n.s.^∗^	2.3 ± 3.9	0.1500	n.s.^∗^	0.5058	n.s.^∗^
REM latency (min)	-6.3 ± 26.4	0.1842	n.s.	-8.4 ± 39.0	0.2816	n.s.	0.8825	n.s.^∗^
N3 latency (min)	-9.4 ± 17.5	0.0285	↓	0.9 ± 3.8	0.7314	n.s.	0.0226	↓
Arousal index	0 ± 0.5	1.0000	n.s.^∗^	0.1 ± 0.4	0.3506	n.s.^∗^	0.5090	n.s.^∗^
Wake after sleep onset (min)	1.4 ± 18.6	0.7694	n.s.^∗^	4.1 ± 6.7	0.1313	n.s.^∗^	0.6286	n.s.^∗^
Percent of sleep efficiency (%)	-0.3 ± 5.0	0.8135	n.s.^∗^	-1.3 ± 2.1	0.1258	n.s.^∗^	0.5123	n.s.^∗^
Time in N1 (min)	-0.6 ± 8.1	0.7912	n.s.^∗^	2.1 ± 5.9	0.3562	n.s.^∗^	0.4300	n.s.^∗^
Time in N2 (min)	-41.2 ± 35.2	0.0002	↓↓	-13.0 ± 20.4	0.0574	n.s.	0.0254	↓
Time in N3 (min)	35.3 ± 24.8	<0.0001	↑↑	1.8 ± 15.2	0.3768	n.s.	0.0011	↑↑
Time in REM (min)	6.7 ± 20.1	0.2156	n.s.^∗^	2.9 ± 17.1	0.6494	n.s.^∗^	0.6504	n.s.^∗^
Percent of N1 (%)	-0.1 ± 1.8	0.8667	n.s.^∗^	0.5 ± 1.3	0.3049	n.s.^∗^	0.4210	n.s.^∗^
Percent of N2 (%)	-9.0 ± 7.1	0.0001	↓↓	-2.2 ± 4.3	0.0987	n.s.	0.0115	↓
Percent of N3 (%)	7.9 ± 5.6	< 0.0001	↑↑	0.8 ± 3.2	0.2628	n.s.	0.0017	↑↑
Percent of REM (%)	1.6 ± 4.0	0.1475	n.s.^∗^	0.9 ± 3.6	0.5095	n.s.^∗^	0.6853	n.s.^∗^

The significant differences between the baseline night and the adaptation night (night 2 compared with night 1) are expressed in **Table 3**. TST and percent of sleep efficiency were significantly increased (*p*-value = 0.0249). REM latency was significant shorter, while N3 latency did not change. The remaining parameters were not significantly different.

**Table 3:** Paired *t*-tests of sleep parameters between night 1 and night 2 of both the experimental and control groups; and independent *t*-tests of the mean difference between the experimental and control groups (comparison of d of night 1 and night 2 between groups; an up arrow indicates an increase or longer duration, and a down arrow indicates a decrease or shorter duration; ^∗^indicates a two-tailed test).

Parameter	Paired *t*-test						Independent *t*-test	
	Experimental group			Control group			*P*-value	Sig.
	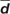 ± SD	*P*-value	Sig.	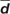 ± SD	*P*-value	Sig.		
Total sleep time (min)	22.6 ± 40.7	0.0249	↑	16.3 ± 38.1	0.2652	n.s.^∗^	0.7237	n.s.^∗^
Sleep latency (min)	-3.4 ± 13.0	0.3225	n.s.^∗^	0.6 ± 3.0	0.5753	n.s.^∗^	0.2640	n.s.^∗^
REM latency (min)	-5.1 ± 93.7	0.0264	↓	-7.6 ± 15.0	0.0973	n.s.	0.0496	↓
N3 latency (min)	2.0 ± 20.2	0.6442	n.s.	-0.1 ± 3.0	0.4542	n.s.	0.6996	n.s.^∗^
Arousal index	-0.1 ± 1.3	0.6976	n.s.^∗^	-0.4 ± 0.9	0.2849	n.s.^∗^	0.6101	n.s.^∗^
Wake after sleep onset (min)	-19.1 ± 38.9	0.0772	n.s.^∗^	-16.9 ± 38.7	0.2558	n.s.^∗^	0.8984	n.s.^∗^
Percent of sleep efficiency (%)	4.7 ± 8.5	0.0249	↑	3.4 ± 7.9	0.2652	n.s.^∗^	0.7237	n.s.^∗^
Time in N1 (min)	0.0 ± 14.5	0.9930	n.s.^∗^	-3.1 ± 10.5	0.4383	n.s.^∗^	0.6013	n.s.^∗^
Time in N2 (min)	16.9 ± 32.0	0.0298	↑	17.2 ± 31.2	0.1628	n.s.^∗^	0.9856	n.s.^∗^
Time in N3 (min)	-4.2 ± 26.7	0.5488	n.s.^∗^	-12.1 ± 18.6	0.1071	n.s.^∗^	0.4661	n.s.^∗^
Time in REM (min)	8.0 ± 24.4	0.2233	n.s.^∗^	19.4 ± 14.5	0.0035	↑	0.2444	n.s.^∗^
Percent of N1 (%)	-0.5 ± 3.8	0.6485	n.s.^∗^	-1.0 ± 2.8	0.3294	n.s.^∗^	0.7126	n.s.^∗^
Percent of N2 (%)	1.0 ± 5.8	0.4994	n.s.^∗^	2.0 ± 4.5	0.2546	n.s.^∗^	0.7028	n.s.^∗^
Percent of N3 (%)	-1.8 ± 5.1	0.2009	n.s.^∗^	-3.5 ± 2.9	0.0060	↓	0.3933	n.s.^∗^
Percent of REM (%)	0.8 ± 4.7	0.5263	n.s.^∗^	3.7 ± 3.0	0.0048	↑	0.1305	n.s.^∗^

#### Control Group

The results show that in each night, participants slept almost entirely in the N2 sleep stage. Significant differences found in the control group are shown in **Table 2**. Comparing the experimental night with the baseline night (night 3 compared with night 2), after the control stimulus (silence) was delivered to the participants during sleep, no significant differences were observed.

Significant differences between the baseline night and the adaptation night (night 2 compared with night 1) are shown in **Table 3**. REM sleep stage significantly increased in terms of both minutes (*p*-value = 0.0035) and percent (*p*-value = 0.0048), while the N3 sleep stage significantly decreased in terms of percent (*p*-value = 0.0060). The remaining parameters did not significantly change.

#### Comparisons Between Groups

The mean differences in these sleep parameters were investigated by independent *t*-tests. The mentioned parameters were compared for differences between nights 2 and 3 of within each group (d between night 2 and night 3 of each group). Significant differences are displayed in **Table 2**. The change in N3 latency between night 3 and night 2 of the experimental group was significantly smaller than that of the control group (*p*-value = 0.0226). The increases in N3 duration between night 3 and night 2 in terms of both minutes (*p*-value = 0.0011) and percent (*p*-value = 0.0017) of the experimental group were significantly greater than those of the control group, while the decreases in N2 duration between night 3 and night 2 in terms of both minutes (*p*-value = 0.0254) and percent (0.0115) of the experimental group were significantly smaller than those of the control group.

Similar comparisons of the mean differences were conducted for night 1 and night 2 between the experimental group and the control group. Significant differences are shown in **Table 3**. Only the mean difference in REM latency between night 1 and night 2 of the experimental group was found to be significantly decreased compared to that of the control group with a *p*-value of 0.0496, which is close to the critical *p*-value of 0.05. The remaining parameters were not significantly different.

### Neural Activity in Each Frequency Band

**FIGURE 4:**
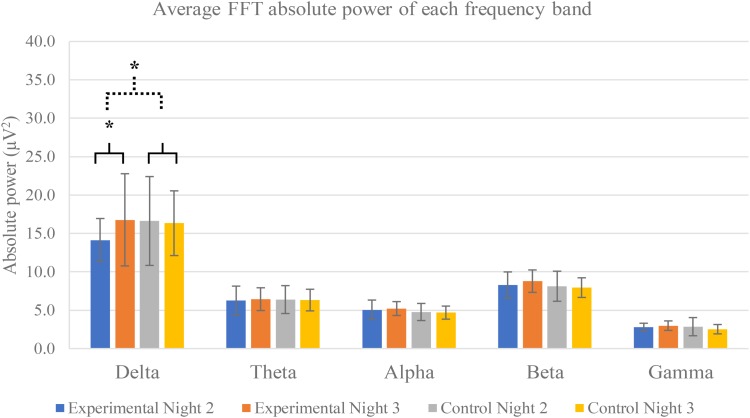
Average FFT absolute power of each frequency band separated by groups and nights. Five frequency bands are exhibited: delta, theta, alpha, beta, and gamma frequency bands. The averages are calculated from all electrode positions including F4, C4, O2, and Cz. Error bars show standard deviations. Solid line brace displays paired *t*-test, and dash line brace displays independent *t*-test. ^∗^Indicates significance.

All frequency bands of neural activity are exhibited in **Figure 4**. FFT absolute power of night 2 and night 3 of both experimental and control groups were calculated to evaluate effect of 3-Hz binaural beat on neural activity.

#### Comparisons Within Groups

**FIGURE 5:**
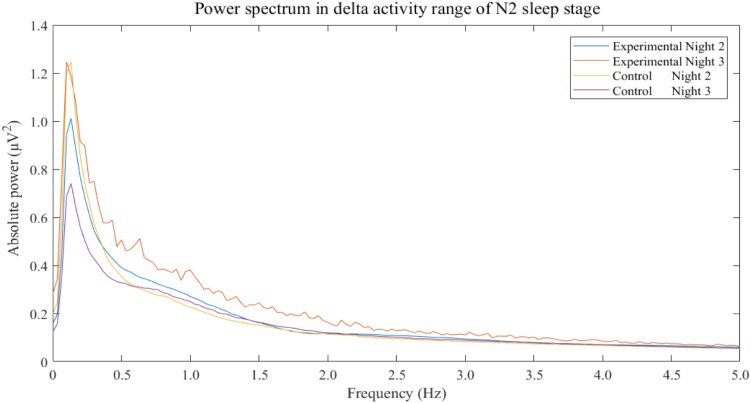
Power spectrum in delta activity range of N2 sleep stage separated by groups and nights. The non-rapid eye-movement stage 2 is labeled as “N2.” Frequencies of more than 5 Hz do not display.

The results show that only delta activity of experimental group was significantly increase between night 2 and night 3 (*p*-value = 0.0293). Other frequency bands of both experimental and control groups were not significant. Power spectral analysis of delta activity during N2 sleep stage of both groups and both nights displayed peaks of activities in range of 0.5–4 Hz of experimental group in night 3 in which the 3-Hz binaural beat was released compared to other nights and control groups (**Figure 5**).

**FIGURE 6:**
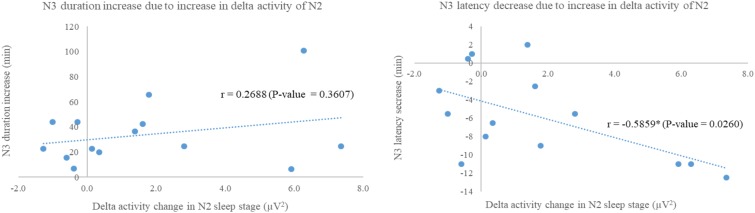
Scatter plots show correlations between delta activity change in N2 sleep stage of night 3 with respect to night 2 of experimental group and N3 duration increase **(Left)**; and N3 latency decrease **(Right)**. Correlation analyses via Fisher transformation were performed only on experimental group due to change in delta activity. The non-rapid eye-movement stages 2 and 3 were labeled as “N2” and “N3,” respectively. ^∗^Indicated significant difference.

As the significances of absolute power of delta activity and extended N3 duration and shorten N3 latency were exhibited only in experimental group, correlations between these factors were performed to evaluate the relationship showing in **Figure 6**. Increase in N3 duration was not significant correlated to increase in delta activity during N2 (*r* = 0.2688, *p*-value = 0.3607), while N3 decrease in N3 latency was negatively significant correlated to increase in delta activity during N2 (*r* = -0.5859, *p*-value = 0.0260).

#### Comparisons Between Groups

The mean differences of these five frequency bands were compared by independent *t*-test and greater significance was found only delta activity between experimental and control groups (*p*-value = 0.0261). Other frequency bands were not found any significance.

#### N3 Sleep Stage Fragmentation

**FIGURE 7:**
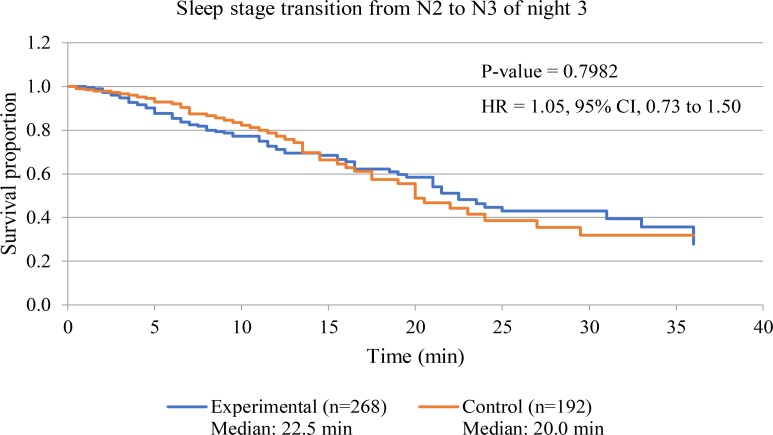
Survival curve of sleep stage transition from N2 sleep stage to N3 sleep stage of night 3 of both experimental and control groups. *P*-value of Log-rank test is displayed. Hazard ratio is labeled as “HR,” and calculated from hazard function of experimental group with respect to that of control group.

**Figure 7** displays survival curve of sleep stage transition from N2 sleep stage to N3 sleep stage of both experimental and control groups to assess sleep fragmentation of experimental group during receiving of 3-Hz binaural beat. Transition time from N2 sleep stage to N3 sleep stage of every transitions from N2 sleep stage was observed in both group. The result shows that no significant difference between experimental group and control group occurred (*p*-value = 0.9782). Therefore, fragmentation of transition from N2 sleep stage to N3 sleep stage in experimental group during 3-Hz binaural beat stimulation was not different from normal sleep of control group.

### Emotional Factors

The emotions of the participants were evaluated by the BRUMS questionnaire each night during the experimental period (three consecutive nights) before going to sleep and after awakening the following morning. Statistical analysis was conducted only fatigue and vigor factors of the experimental night to evaluate sleep disturbance effect of 3-Hz binaural beat.

#### Comparisons Within Groups

The results showed that almost items of fatigue factors decreased after waking up in both experimental and control group, and two items of vigor factor increased after waking up in experimental group (**Table 4**).

**Table 4:** Paired *t*-test of the BRUMS scores only fatigue and vigor factors between before sleep and after waking for night 3 of both the experimental and control groups; and independent *t*-tests of the mean difference between the experimental and control groups, which were only performed for significant differences shown by the paired *t*-test (comparison of d for night 3 between the groups; up arrow indicates an increase, down arrow indicates a decrease).

Factor	Emotion	Paired *t*-test						Independent *t*-test	
		Experimental group			Control group			*P*-value	Sig.
		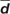 ± SD	*P*-value	Sig.	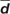 ± SD	*P*-value	Sig.		
Fatigue	Worn out	-0.40 ± 0.91	0.0554	n.s.	-0.38 ± 0.52	0.0398	↓		
	Exhausted	-0.47 ± 0.99	0.0447	↓	-0.63 ± 0.52	0.0056	↓	0.2827	n.s.
	Sleepy	-0.67 ± 1.05	0.0136	↓	0.13 ± 0.99	0.3659	n.s.	0.0512	n.s.
	Tired	-0.67 ± 1.29	0.0326	↓	-1.00 ± 0.53	0.0006	↓↓	0.2312	n.s.
Vigor	Lively	-0.13 ± 1.30	0.3488	n.s.	-0.75 ± 1.16	0.0557	n.s.		
	Energetic	0.53 ± 1.41	0.0822	n.s.	0.25 ± 0.89	0.2256	n.s.		
	Active	0.73 ± 0.70	0.0006	↑	-0.38 ± 0.92	0.1425	n.s.	0.0064	↑
	Alert	0.53 ± 0.92	0.0203	↑	0.25 ± 0.89	0.2256	n.s.	0.2197	n.s.

#### Comparison Between Groups

Only significant differences in experimental group were greater analysis. From **Table 4**, the significant difference of the mean difference between before sleep and after waking up between both group revealed that only active was found to significantly increase. The remaining emotions did not show any significant changes. Therefore, the increase in active feelings of the experimental group was greater than that of the control group.

## Discussion

Three-Hz binaural beat on 250-Hz carrier tone cloud modulate neural activity by inducing delta activity during sleep and exhibited effects on sleep stages by increase in N3 sleep stage with decrease in N2 sleep stage, shorten N3 latency, and did not disturb sleep continuity indicated by arousal index and WASO without sleep fragmentation induction.

The 3-Hz binaural beat on a 250-Hz carrier tone seemed to directly affect the N3 and N2 sleep stages. Time in N3 sleep was extended during the experimental night of the experimental group compared with the baseline night of the same group. This increased N3 duration was induced by the 3-Hz binaural beat, as was clearly observed by the comparison of the change in the control group at the same time-point, night 3 compared with night 2. This comparison was conducted to confirm that this effect was indeed the effect of the stimulus, not the effect of habituation to the surrounding environment. The duration of N3 sleep was increased by the 3-Hz binaural beat not only in terms of time but also normalized time, as the percent of TST in the N3 stage. Because of different sleep onsets, the percent of TST in each stage should be used to normalize the sleep time in each stage for comparisons. The results suggest that the increase in the duration and percent of N3 sleep stage of the experimental group on the experimental night compared with other nights and groups was an effect of the 3-Hz binaural beat. With these procedures of comparison, N3 latency was shorten and N2 duration was decreased by the effects of 3-Hz binaural beat without sleep discontinuity revealed by WASO.

In other words, N3 duration was extended with shortening N2 duration, and N3 latency was shorten potentially resulting from 3-Hz binaural beat stimulation during sleep in which comparisons were performed within the experimental group, and the results were confirmed indeed effect of the stimulus, not the habituation to the surrounding environment, by comparisons of the changes of the control group.

The changes in N3 sleep stage during the experimental night was in accordance with the increase in delta activity during that night. Delta activity contains frequency of 3 Hz so 3-Hz binaural beat is in range of delta activity. According to binaural beat effects which can entrain neural synchronization, several studies have reported this entrainment effect on other frequency bands as mentioned (Gray and Singer, 1989; Llinas and Pare, 1991; Gray, 1999; Vanderwolf, 2000; Takahashi et al., 2005; Lagopoulos et al., 2009; Jirakittayakorn and Wongsawat, 2017a,b) but investigation of the enhancement effect of delta activity during sleep have been lacked. However, this study was shown that power of delta activity seemed to be induced by 3-Hz binaural beat and the increase in delta activity was related to the increase in N3 sleep stage. Moreover, spectral components of delta activity which are in range of 0.5 Hz to 4 Hz of experimental group in experimental night, night 3, were clearly over compared to baseline of the same group and those of control group (**Figure 5**). The power spectrum of delta activity during N2 sleep stage was closely reflect the effect of the stimulus on EEG signal due to criteria of stimulation. The results showed higher values and peaks in every delta component which potentially indicated that the 3-Hz binaural beat affected delta activity EEG signal during sleep.

In order to reveal how does the 3-Hz binaural beat affect to N3 sleep stage, correlation analyses between increase in delta power during N2 sleep stage and either increase in N3 duration or decrease in N3 latency were performed pairwise. The significance was found only relationship between increase in delta power during N2 and decrease in N3 latency with a strong negative correlation which reflected that the more delta power during N2 increase, the shorter N3 latency. However, increase in N3 duration showed slight positive trend related to increase in delta power during N2 sleep stage. With these, the 3-Hz binaural beat seemed to increase N3 sleep stage by shorten N3 latency after that N3 duration trended to increase, spontaneously. Some explanations were given in recent study that a unidirectional increase in firing rate related to the stimulus of cortical neurons during UP-states without decreasing the firing rate during DOWN-states (Reato et al., 2013; Saebipour et al., 2015), and lasting of after-effect of the transcranial alternating current stimulation on EEG endogenous power, especially on low frequency power (Neuling et al., 2013). These explanations seemed to be used to explain our findings as the 3-Hz binaural beat could be used to induce delta activity during N2 sleep stage leading to shorten N3 latency. After N3 sleep stage occurred, the neurons firing during N3 sleep stage continuously fired in delta activity due to homeostasis without decrease in firing rate of delta activity until transition out to the other sleep stages.

The interesting role of slow wave sleep or N3 sleep stage is memory consolidation (Walker, 2009a). The consolidation process is important for memory stabilization after receiving of information into the brain which occurs during learning phase. Sleep is also associated with all processes including memory reconsolidation, memory enhancement, preventing memory destabilization, and deterioration (Walker and Stickgold, 2006). Non-emotional, fact-based memories or episodic memories is associated with presence and integrity of slow wave sleep (Backhaus et al., 2006). An experiment reported that primary insomnia of which slow wave sleep decreased showing less memory consolidation than normal (Backhaus et al., 2006), and normal healthy people with the age of over 30 years old whose polysomnography exhibited decrease in slow wave sleep during the night were found decrease in memory consolidation (Backhaus et al., 2007).

The process of memory formation and consolidation occurs by which co-ordination between neocortex and hippocampus of the brain (Wiltgen et al., 2004; Frankland and Bontempi, 2005). Fragments of memory are formed at several regions and binding together of these fragments is generated within this network. This stage is so-called learning phase. Gamma oscillation plays an important role to combine them together (Jirakittayakorn and Wongsawat, 2017b). During consequent period, sleep period in slow wave sleep stage or N3 sleep stage, the memory receiving in learning phase is reactivated again (Walker, 2009b), and consolidation process occurs. The neural oscillation of slow wave sleep has origination at neocortex (Buzsaki, 1996).

The relationship of memory formation and consolidation and slow wave sleep potentially indicates that our study can be widely implied, nowadays. Because of modern lifestyle, slow wave sleep seems to be disturbed. Stress in modern lifestyle has interacted with sleep architecture and other factors (Buckley and Schatzberg, 2005) leading to impairment of quality of life (Hirotsu et al., 2015) by reduction of slow wave sleep, REM sleep, delta power (Rolls et al., 2010), and sleep efficiency including increase in awakening (Kim and Dimsdale, 2007). In addition, chronic work overload is also the cause of slow wave sleep and REM sleep decreases (Gonnissen et al., 2013). Because stress in daily life perturb healthy sleep (Mason, 1968) by decrease in slow wave sleep. Slow wave sleep play an important role in memory formation and consolidation so our finding can be utilized to improve sleep quality and memory by inducing of slow wave sleep.

Besides memory consolidation, memory reconsolidation is another process related to slow wave sleep. Memory reconsolidation process is explained that once storage memory is retrieved from a trace after storage, it becomes labile or unstable memory once again. That unstable memory which is sensitive to disruption is reconsolidated to the storage after reactivation (Alberini and LeDoux, 2013). The function of memory reconsolidation is still unclear (Walker and Stickgold, 2006) but it has been hypothesized into to two separate thoughts. The first one is considered to be memory updating of incremental learning following re-exposure to an experience similar to the first learning. The second one is considered to be memory strengthening and weakening depending on an experience that retrieve the learned experience (Alberini and LeDoux, 2013; Bisaz et al., 2014). However, due to the fragile state in a limited time of that memory, some disruptions can lead to change in some part of that memory before reconsolidation process. After reconsolidation process, the changed memory is re-stored to the storage. With this phenomenon, our finding seems to be one potential tool of several tools for memory reconsolidation in memory reconsolidation study. By increase in N3 sleep stage or slow wave sleep leading to improve the memory reconsolidation process. During unstable memory or labile state, if that memory is interfered by some stimulus, it trends to be adjusted or modulated by that stimulus. After memory reconsolidation process is completed due to quality sleep, the changed memory is stored instead of the old one. Potentially, these procedures can become session training either for strengthening or weakening the targeted memory. One study, which has been in line with our postulation, showed that fear memory can be abolished by a stimulus which was similar to the stimulus that induced fear memory (James et al., 2015) during reconsolidation process. Further studies are required for this postulation but improve quality of sleep due to increase in slow wave sleep is like to be modulated by the 3-Hz binaural beat.

Some studies have attempted to modulate sleep stage by inducing of slow wave sleep and indicated that recognition memories have been increased due to increase of slow wave sleep which were related to this study. One study reported that memory can be greater recalled in napping participant with slow wave sleep than without slow wave sleep (Takashima et al., 2006). Odor stimulus was used in one study (Rasch et al., 2007) stating that memory to card pair increased after odor stimulation was performed to participants during slow wave sleep compared with odorless group. Moreover, the researchers found that odor stimulation during REM sleep and wakefulness did not affect memory. However, the studies of inducing of slow wave sleep have exhibited the results consisting to our results in which slow wave sleep can be induced. Transcranial magnetic stimulation (TMS) (Huber et al., 2007; Massimini et al., 2007) and transcranial direct current stimulation (tDCS) (Marshall et al., 2004, 2006) have been used to induce slow wave sleep. The TMS of 5 Hz pulse was delivered to participants during wakefulness finding that slow wave activity rose up during sleep (Huber et al., 2007), while the TMS of <1 Hz pulse delivered during sleep was also raised slow wave activity up (Massimini et al., 2007). The uses of tDCS expressed enhancement of memory (Marshall et al., 2004) and slow wave sleep (Marshall et al., 2006). The tDCS was delivered to participants during slow wave sleep finding that memory was improved (Marshall et al., 2004), while the tDCS of 0.75 Hz delivered in N2 sleep stage can induce slow wave sleep and improve the memory recall (Marshall et al., 2006). These studies were firmly corresponding to our finding. However, our finding provided a novel investigation of 3-Hz binaural beat on sleep architecture during sleep.

To clarify that these effects, extended N3 duration and percent, shortened N3 latency, and shortened N2 duration and percent, were the effects of the stimulus and not other factors, all baseline nights should be the same. The results indicated that participants in both the experimental group and the control group were not deprived of sleep during the adaptation night (the first night) and that the adaptation night only allowed the participants to adapt to the new environment. TST, percent of sleep efficiency, WASO, and time in each stage including the percent of each sleep stage did not differ. N3 latency and REM latency were also not different, which also offers potential supporting evidence, because sleep in every night is affected by the sleep history of the previous night. If sleep deprivation occurs, slow wave sleep or N3 sleep stage and REM sleep stage are observed less (Hediger, 1980; Carskadon and Dement, 2005). Thus, the baseline nights of the experimental and control groups did not differ.

Sleep stages and other sleep parameters indicated that the 3-Hz binaural beat on a 250-Hz carrier tone can be potentially used to modulate sleep stage. Shortened N2 duration, extended N3 duration, and shortened N3 latency were presented after receiving the 3-Hz binaural beat on a 250-Hz carrier tone during sleep. Furthermore, the results also suggested that an auditory stimulus can be utilized to modulate sleep stage without disturbing sleep, which is in agreement with some studies on auditory pathways and sleep (Drucker-Colin et al., 1983; Arankowsky-Sandoval et al., 1986; Vazquez et al., 1998; Amici et al., 2000) and with some studies on stimulation during sleep discussed above (Huber et al., 2007; Massimini et al., 2007; Marshall et al., 2004, 2006). Sleep disturbance can be observed by increases in N2 duration and the awake stage (Terzano et al., 1990), contrary to the results. Moreover, arousal index did not differ between experimental and control groups, so the 3-Hz binaural beat did not induce arousal during sleep. However, emotions were evaluated to subjectively identify any sleep disturbances due to receiving the 3-Hz binaural beat. Fatigue factor of BRUMS reveals exhaustion of the participants after waking up, the results indicated that all participants of did not feel any fatigue after waking up. While vigor factor reveals powerfulness, the results indicated that participants in experimental group were active and alert than control group. Therefore, the stimulus did not disturb the sleep of all participants.

In addition to sleep disturbance which had to be concerned during sleep stimulation, sleep fragmentation should be evaluated to clarify continuity of sleep stage. As the stimulation released to participants in this study, transition stage from N2 sleep stage to N3 sleep stage was kept an eye on due to auditory stimulation at N2 sleep stage. Three-Hz binaural beat can induce N3 sleep stage from N2 sleep stage but after auditory stimulation was over, sleep stage may become N2 sleep stage, so sleep fragmentation was a concerned factor. The results showed that during experimental night of experimental group and that of control group, transition stage from N2 sleep stage to N3 sleep stage did not differ. This result from survival analysis indicated that 3-Hz binaural beat did not cause sleep fragmentation.

These findings suggested that the 3-Hz binaural beat on a 250-Hz carrier tone can be used to modulate sleep stage by decreasing the latency to the N3 stage, extending the N3 duration, and reducing the N2 duration without sleep disturbance and sleep fragmentation, while increasing the quality of sleep in this study is also associated slow wave sleep in which is related to memory consolidation and other regulation of the body; and the 3-Hz binaural beat can enhance power of delta activity during sleep. Other effects should be further study, but with this novel insight, it seems to imply to several applications using the binaural beat as stimulus to modulate the brain during sleep. Surprisingly, after the experimental period had finished, some participants in the experimental group asked the researchers about the training session for sleep quality improvement, because they reported that they felt excellent after waking up; however, they had remained blinded to the fact that they were part of the experimental group.

### Limitations

This study evaluated the acute effect of a binaural beat stimulus in a single night. Long-term exposure and habituation should be further investigated. The carrier tone utilized in this study was only 250 Hz, and other carrier tones should be further investigated because different brain processes are affected by different carrier tones. Another beat frequency should be used to compare effect on sleep with 3-Hz, binaural beat. Moreover, event-related potential (ERP) should be further analyzed at the time of stimulation occurs to evaluate the brain response to auditory sudden change, transition from environmental sound intensity to stimulus intensity, but the methodology should be time-fixed. Cross-over design of the experiment should be conducted to more clarify effects of the stimulus does not depend on groups of participants.

### Suggested Application

One interesting application of using 3-Hz binaural beat besides induction of N3 sleep stage was investigations of long-term memory function due to exposure of 3-Hz binaural beat as training session for improvement of sleep quality. With this investigation, long-term exposure to the 3-Hz binaural beat can be observed and effect of habituation can be clarified.

## Conclusion

A 3-Hz binaural beat on a 250-Hz carrier tone can be used to modulate neural activity by enhancement of power of delta activity; and to modulate sleep stage by decreasing N2 duration, inducing the N3 stage, and increasing N3 duration without sleep disturbance and sleep fragmentation.

## Author Contributions

NJ generated the protocol and conducted the experiments. YW proved and edited the protocol.

## Conflict of Interest Statement

The authors declare that the research was conducted in the absence of any commercial or financial relationships that could be construed as a potential conflict of interest.
